# Supplementary material for: COZOID: contact zone identifier for visual analysis of protein-protein interactions
Source: BMC Bioinformatics. 2018 Apr 6;19:125. doi: 10.1186/s12859-018-2113-6 (PMC5889581; doi:10.1186/s12859-018-2113-6)
Supplement: Supplementary file 2 — Software build. Executable binary file of the software tool. (ZIP 118,784 kb) [file 12859_2018_2113_MOESM2_ESM.zip › cozoid/platform/docs/testng-6.5.1-javadoc.zip!/org/testng/annotations/package-tree.html]

org.testng.annotations Class Hierarchy (TestNG)


---


|  |  |  |
| --- | --- | --- |
| **Overview** \| **Package** \| Class \| **Tree** \| **Deprecated** \| **Index** \| **Help** | |  |
| **PREV**   **NEXT** | **FRAMES**    **NO FRAMES**     **All Classes** |  |


---


## Hierarchy For Package org.testng.annotations

**Package Hierarchies:**: All Packages

---

## Interface Hierarchy

- org.testng.annotations.**IAnnotation**
  - org.testng.annotations.**IDataProviderAnnotation**- org.testng.annotations.**IExpectedExceptionsAnnotation**- org.testng.annotations.**IObjectFactoryAnnotation**- org.testng.annotations.**IParameterizable**
          - org.testng.annotations.**IFactoryAnnotation** (also extends org.testng.internal.annotations.IDataProvidable)- org.testng.annotations.**ITestOrConfiguration**
              - org.testng.annotations.**IConfigurationAnnotation**- org.testng.annotations.**ITestAnnotation** (also extends org.testng.internal.annotations.IDataProvidable)
          - org.testng.annotations.**IParametersAnnotation**
  - org.testng.internal.annotations.IDataProvidable
    - org.testng.annotations.**IFactoryAnnotation** (also extends org.testng.annotations.IParameterizable)- org.testng.annotations.**ITestAnnotation** (also extends org.testng.annotations.ITestOrConfiguration)

## Annotation Type Hierarchy

- org.testng.annotations.**BeforeMethod** (implements java.lang.annotation.Annotation)- org.testng.annotations.**Test** (implements java.lang.annotation.Annotation)- org.testng.annotations.**AfterMethod** (implements java.lang.annotation.Annotation)- org.testng.annotations.**TestInstance** (implements java.lang.annotation.Annotation)- org.testng.annotations.**NoInjection** (implements java.lang.annotation.Annotation)- org.testng.annotations.**AfterGroups** (implements java.lang.annotation.Annotation)- org.testng.annotations.**AfterClass** (implements java.lang.annotation.Annotation)- org.testng.annotations.**BeforeSuite** (implements java.lang.annotation.Annotation)- org.testng.annotations.**ObjectFactory** (implements java.lang.annotation.Annotation)- org.testng.annotations.**AfterSuite** (implements java.lang.annotation.Annotation)- org.testng.annotations.**Configuration** (implements java.lang.annotation.Annotation)- org.testng.annotations.**BeforeTest** (implements java.lang.annotation.Annotation)- org.testng.annotations.**Optional** (implements java.lang.annotation.Annotation)- org.testng.annotations.**AfterTest** (implements java.lang.annotation.Annotation)- org.testng.annotations.**BeforeClass** (implements java.lang.annotation.Annotation)- org.testng.annotations.**Factory** (implements java.lang.annotation.Annotation)- org.testng.annotations.**ExpectedExceptions** (implements java.lang.annotation.Annotation)- org.testng.annotations.**Parameters** (implements java.lang.annotation.Annotation)- org.testng.annotations.**DataProvider** (implements java.lang.annotation.Annotation)- org.testng.annotations.**Listeners** (implements java.lang.annotation.Annotation)- org.testng.annotations.**BeforeGroups** (implements java.lang.annotation.Annotation)- org.testng.annotations.**Guice** (implements java.lang.annotation.Annotation)

---


|  |  |  |
| --- | --- | --- |
| **Overview** \| **Package** \| Class \| **Tree** \| **Deprecated** \| **Index** \| **Help** | |  |
| **PREV**   **NEXT** | **FRAMES**    **NO FRAMES**     **All Classes** |  |


---
